# Midterm MRI Follow-Up of Untreated Enchondroma and Atypical Cartilaginous Tumors in the Long Bones

**DOI:** 10.3390/cancers13164093

**Published:** 2021-08-13

**Authors:** Claudia Deckers, Jacky W. J. de Rooy, Uta Flucke, H. W. Bart Schreuder, Edwin F. Dierselhuis, Ingrid C. M. van der Geest

**Affiliations:** 1 Department of Orthopedics, Radboud University Medical Center, 6525 GA Nijmegen, The Netherlands; bart.schreuder@radboudumc.nl (H.W.B.S.); Edwin.Dierselhuis@radboudumc.nl (E.F.D.); Ingrid.vanderGeest@radboudumc.nl (I.C.M.v.d.G.); 2 Department of Radiology, Nuclear Medicine and Anatomy, Radboud University Medical Center, 6525 GA Nijmegen, The Netherlands; Jacky.deRooy@radboudumc.nl; 3 Department of Pathology, Radboud University Medical Center, 6525 GA Nijmegen, The Netherlands; Uta.Flucke@radboudumc.nl

**Keywords:** chondrosarcoma, watchful waiting, magnetic resonance imaging, bone neoplasms

## Abstract

**Simple Summary:**

Over the last decade the incidence of enchondroma and atypical cartilaginous bone tumors (ACTs) increased enormously. Management of these tumors in the long bones is shifting towards active surveillance, as negative side effects of surgical treatment seem to outweigh the potential benefits. To support development of evidence-based guidelines for active surveillance, we studied the natural course of enchondroma and ACTs in the long bones. In this study, MRI analysis of 128 cases was performed with a minimum interval of 24 months between baseline and last MRI. Our data showed that the majority of the cartilaginous tumors (87%) remained stable or showed regression on MRI. Only 13% showed some progression on MRI, although none of the tumors developed characteristics of high-grade chondrosarcoma. Based on our results, active surveillance is considered safe for enchondroma and ACTs of the long bones, and follow-up schemes should be tailored on natural course.

**Abstract:**

Management of atypical cartilaginous tumors (ACTs) in the long bones is shifting towards active surveillance to avoid unnecessary surgeries. The frequency and duration of active surveillance for these tumors is unclear as there is little knowledge of its biological behavior. In this retrospective study, we examined the natural course of enchondroma and ACTs through active surveillance. A total of 128 central cartilaginous tumors, located in the long bones, with a minimum interval of 24 months between baseline and last MRI were included. MRI characteristics (e.g., size, scalloping, fat entrapment) were scored and tumors were classified according to the changes between MRIs. Mean follow-up of this study was 50 months, range = 25–138 months. The majority of the cartilaginous tumors (87%) remained stable (*n* = 65) or showed regression (*n* = 46) on MRI. A total of 87% of the cases that developed tumor regression presented with entrapped fat at diagnosis. Only 13% (*n* = 17) showed some progression on MRI, although none of the tumors developed characteristics of high-grade chondrosarcoma. Based on our results, active surveillance is considered safe for enchondroma and ACTs of the long bones. We propose active surveillance for all asymptomatic enchondroma or ACTs in the long bones irrespective of tumor size, and follow-up schemes should be tailored on natural course.

## 1. Introduction

Nowadays central cartilaginous bone tumors are reported as the most common primary bone tumor. Enchondroma are benign cartilaginous tumors, whereas chondrosarcoma are malignant cartilaginous tumors. Chondrosarcomas are divided into grades ranging from 1 to 3 which correlate with aggressiveness and; therefore, with local recurrence rate, metastatic potential and the disease specific potential. Since chondrosarcoma grade 1 of the long bones rarely metastasizes and show no signs of local malignant behavior, the WHO decided in 2013 to change the classification of chondrosarcoma grade 1 (CS1) from malignant into locally aggressive [1]. In the latest 2020 WHO classification, a clear distinction between CS1 in the axial skeleton and appendicular skeleton was made [2]. Tumors in the axial skeleton are still termed CS1, reflecting the more aggressive local behavior and poorer clinical outcome of tumors at these sites, whereas CS1 in the appendicular skeleton are now termed atypical cartilaginous tumors (ACTs). Typical for cartilaginous tumors are the popcorn-like calcifications and the lobulated contours. Due to the numerous similarities of enchondroma and ACTs on imaging, differentiating these tumors is problematic [3,4,5]. High-grade chondrosarcoma is recognized by aggressive radiologic features such as bone destruction and soft tissue extension.

Several studies have shown a rise in incidence of chondrosarcoma over the last decades, which is mainly caused by ACTs and might be explained by a simultaneous increase of MRI usage, resulting in more incidental findings [6,7]. Similarly, the incidence of enchondroma increased over the last decades [6]. Orthopedic oncologists and radiologists are more frequently confronted with the diagnostic dilemma of differentiating enchondroma and ACT [3,4,5]. The slight increase in incidence of high-grade chondrosarcoma (grade 2, 3 and dedifferentiated chondrosarcoma) is much lower than the increase in incidence of ACTs and enchondroma [6,7]. Based on recent studies on the incidence of cartilaginous tumors, the risk of transformation of atypical cartilaginous tumors (ACTs) to high-grade chondrosarcoma (HGCS) is assumed to be <1%, instead of the previously reported risk of 2.5–6% [6,7].

Due to these new insights, the most recent literature on cartilaginous tumors shifts towards active surveillance for ACTs in the long bones to avoid unnecessary surgeries [8,9,10,11]. Several authors have studied active surveillance for central cartilaginous tumors in the long bones without aggressive imaging characteristics (e.g., cortical destruction, soft tissue expansion) [12,13,14,15]. Omlor et al. showed that active surveillance of enchondroma and ACTs has benefits in clinical and functional outcomes compared to surgical treatment [15]. However, the frequency and the duration of active surveillance for these tumors is unclear, as there is little knowledge of their natural course. The few studies that describe the natural course of enchondroma and ACTs are limited by small numbers of patients and short follow-up [12,14,16]. Important to mention is that none of these studies described transformation to HGCS. Overall, active surveillance seems appropriate for central cartilaginous tumors in the long bones without aggressive characteristics.

The aim of this study is to examine the natural course of central cartilaginous tumors located in the long bones without aggressive imaging characteristics. As magnetic resonance imaging (MRI) is the imaging method of choice for central cartilaginous tumors [17], we analyzed MRI characteristics of enchondroma and ACTs, in which we refrained from surgery.

## 2. Methods

**Figure 1:**
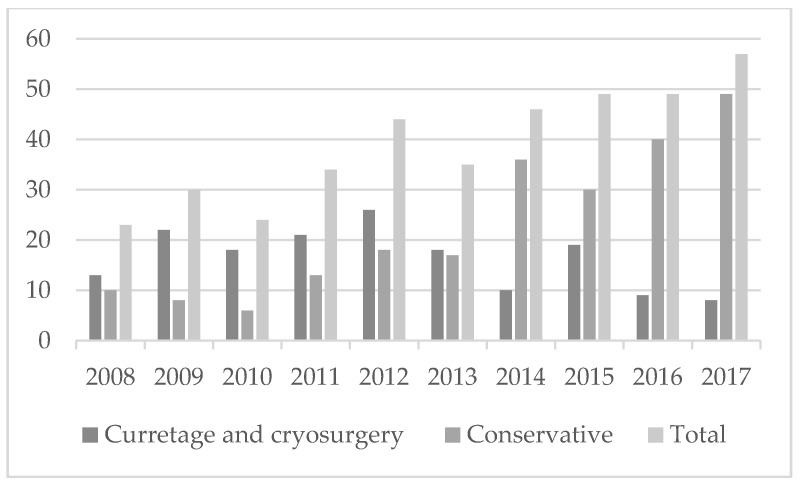
Number of cartilaginous tumors without aggressive characteristics located in the long bones treated at our institution between 2008 and 2017.

A retrospective cohort study was performed on all patients diagnosed between 2008 and 2017 with central cartilaginous tumors in the long bones without aggressive imaging characteristics. In total, 391 cartilaginous tumors without aggressive imaging characteristics were located in the long tubular bones, of which 227 tumors underwent active surveillance instead of surgical removal (See Figure 1).

For the purpose of this study, patients were included if they met the following criteria: (1) A central cartilaginous tumor located in the long tubular bones, (2) under active surveillance, and (3) who underwent at least two MRIs of the tumor with a minimum time interval of 24 months. Patients with Ollier’s disease or Maffucci syndrome were excluded. Previously biopsied tumors were also excluded as this could interfere with MRI interpretation.

**Figure 2:**
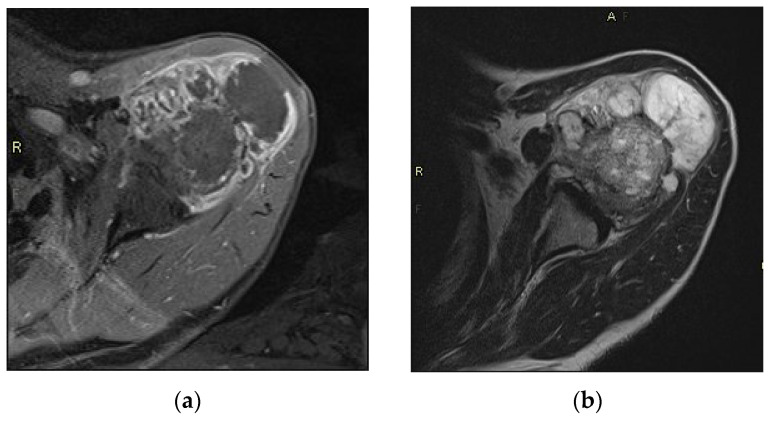
(**a**,**b**): Axial CE (contrast enhanced) TSE T1-weighted and axial SE T2-weighted image of the proximal humerus of the same patient: Extensive cortical break-through with soft tissue mass in a 66-year-old male with histopathologically proven dedifferentiated chondrosarcoma.

We did not differentiate between enchondroma and ACT due to the similarities of these tumors when located in the long tubular bones [3,4,5]. After referral to our hospital, physical examination was performed and all imaging was reviewed by experienced musculoskeletal radiologists and, if necessary, additional imaging (i.e., X-ray and/or MRI) was performed [16]. Active surveillance was advised when neither invalidating pain nor any of the following radiological signs of malignancy were seen: cortical destruction, presence of soft tissue mass, moth-eaten, permeative or extensive osteolysis, multi-lamellar or aggressive periosteal reaction [16,18,19]. Figure 2 shows an example of an excluded patient with radiological signs of malignancy. Patients who accepted the active surveillance were followed-up with MRI after 6 months. If MRI showed none of the previously described malignant characteristics or excessive tumor growth, active surveillance was continued. Patients were educated on their diagnosis and instructed to contact our hospital in case of new or increased pain. When invalidating pain or progression of the tumor on MRI (i.e., tumor growth, malignant characteristics) occurred, or when the patients revised their choice for conservative therapy, patients were surgically treated. Our surgical method consists of curettage and cryosurgery, filling of the void with cement or bone graft, and in some cases prophylactic plating [20,21]. Curettage material was reviewed by experienced pathologists and histological diagnosis was made according to established criteria [1,22]. All operated cases were regularly monitored after surgery, consisting of physical examination and conventional radiology. In cases of clinical or radiological suspicion of local recurrence, additional MRI was performed.

### 2.1. MRI Analysis

At our institution all MRIs were performed on a 1.5T scanner, and intravenous gadolinium was standardly used. Most patients were referred to our tertiary center with a baseline MRI, often performed without gadolinium in the referring hospital. All MRIs analyzed in this study were performed between 2008 and 2020. An experienced senior musculoskeletal radiologist (JdR) scored the following tumor characteristics: craniocaudal size, location, presence of endosteal scalloping, peritumoral edema, fat entrapment, ring and arc enhancement, calcifications, and replacement of cartilaginous lobules by fatty marrow.

Tumor size was measured as the largest craniocaudal dimension on coronal or sagittal images. To account for possible measurement error, only a size difference of ≥3 mm was considered progression or regression. Endosteal scalloping defined as loss of inner cortical bone was evaluated on axial images. The degree of scalloping was not quantified, scalloping was scored as increased/decreased when there was increased/decreased circumferential loss of inner cortical bone compared with the baseline MRI. Fat entrapment was defined as presence of intralesional foci of high signal intensity on T1-weighted images, correlating with fat within the cartilage tumor, as described by Vanel et al. [23]. Ring and arc enhancement could only be scored when contrast enhanced MRI was available. Loss of enhancement might be attributed to increased fat entrapment, increased calcification or use of different imaging techniques resulting in different contrast uptake. Fatty replacement was only scored on the last MRI and defined as replacement of cartilaginous lobules by normal fatty marrow.

After scoring of the tumor characteristics at baseline and last MRI, tumors were classified as regression (R), progression (P), or no change (NC). Tumors were classified as regression when one or more of the following changes occurred: (1) Decreased tumor size (≥3 mm), (2) decreased scalloping, (3) increased calcification, (4) increased fat entrapment or (5) replacement of cartilaginous lobules by fatty marrow. Tumors were classified as progression when one or more of the following characteristics were scored: (1) Increased tumor size (≥3 mm), (2) increased scalloping or (3) loss of calcification.

### 2.2. Statistics

Analysis of the data was performed using IBM SPSS statistics for Windows (version 25). All continuous variables were visually inspected and tested for normality by the Shapiro-Wilk test.

After MRI analysis, three groups were defined (R, P and NC) and the differences at first presentation between these groups were assessed. Kruskal–Wallis and Mann–Whitney U were used for continuous variables. For categorical variables, Chi-squared or Fisher’s exact tests depending on expected frequency in cells were used. *p*-value < 0.05 was considered statistically significant.

## 3. Results

In total, 128 tumors in 124 patients (47 male, 77 female) met the inclusion criteria for this retrospective study. Mean age at diagnosis was 52 years (range 20–76 years). Patient demographics are reported in Table 1. As our hospital is a tertiary care referral center, 73% of the cases were first diagnosed in another hospital. Mean time of conservative follow-up since initial diagnosis is 50 months (range = 25–138 months).

### 3.1. Symptoms at Presentation

The majority of the lesions were incidental findings (125 cases; 97.7%), whereas two cases (1.6%) were referred with pain that could not be explained by adjacent pathology, and for one case the referral indication could not be retrieved from the clinical charts. Most incidental findings (22.4%) were found after patients presented themselves with knee complaints not related to the tumor (e.g., osteoarthritis, meniscal tears). Some of the other reasons for imaging were trauma (14.1%), shoulder complaints not related to the tumor (14.8%), cancer staging imaging studies (8.6%) and rheumatoid arthritis screening (7.0%). Only two patients (1.6%) reported pain that could not be explained by adjacent pathology and was; therefore, probably related to the tumor. In both cases pain complaints were transient and active surveillance was continued. Seven cases developed new pain complaints during follow-up. In six out of seven cases, physical examination and imaging showed other causes (e.g., bursitis, osteoarthritis) for the pain. In one case no other cause was found but the pain was transient and active surveillance was continued.

### 3.2. MRI Analysis

A total of 65 cases showed no change (NC), 46 regression (R) and 17 progression (P). MRI characteristics are summarized in Table 2. One case presented with peritumoral edema, which could be explained by a recent fracture and subsequently diminished on the last MRI. Of the 46 cases in the regression group, 37 cases showed fatty replacement, 39 increased fat entrapment, five decreased scalloping, 11 increased calcifications and 17 decrease of tumor size (mean tumor decrease 8 mm; range 3–30 mm). In the majority of the cases (83%), a combination of two or more MRI changes related to regression were seen. Two patients of the regression group showed tumor growth (3 and 10 mm) prior to development of signs of regression (increased fat entrapment, fatty replacement). All 17 cases with progression of the tumor showed increased tumor size over time (median tumor growth 5 mm; range 3–23 mm). Only eight of the 17 cases showed tumor growth over time of more than 5 mm. Two cases showed loss of ring and arc enhancement in addition to 6 and 23 mm tumor growth (respectively, 62 and 51 months follow-up). The tumor with 23 mm growth was removed and histopathologically diagnosed as ACT (Table 3). The other patient remains under active surveillance as tumor growth developed gradually and the patient has no pain complaints. None of the cases included in this study showed any aggressive MRI characteristics often seen in HGCS (e.g., cortical destruction or soft tissue mass extension) during follow-up.

In total, six of the 128 cases included in this study were operated (Table 3), either due to tumor growth (*n* = 5) or osteoarthritis and total knee arthroplasty was combined with curettage of the lesion (*n* = 1). None of the operated cases transformed into HGCS.

## 4. Discussion

Changes in the WHO classification from chondrosarcoma grade 1 of the long bones to atypical cartilaginous tumor (ACT), together with the recent insight of low transformation risk of ACTs (<1%), has resulted in a more conservative treatment approach of ACTs in literature [1,2,6,7]. Many authors have questioned if the negative side effects of surgical treatment outweigh the potential benefits and; therefore, proposed active surveillance [6,8,10,12,14,15,16]. To support development of evidence-based guidelines for active surveillance, we studied the natural course of enchondroma and ACTs in the long bones. In this study, MRI analysis of 128 cases was performed with a minimum interval of 24 months between baseline and last MRI. The majority of the cartilaginous tumors (87%) remained stable or showed regression on MRI. Only 13% showed some progression on MRI, although none of the tumors developed characteristics of high-grade chondrosarcoma (HGCS). Based on our results, active surveillance is considered safe for enchondroma and ACTs of the long bones, and follow-up schemes should be tailored on natural course.

**Figure 3:**
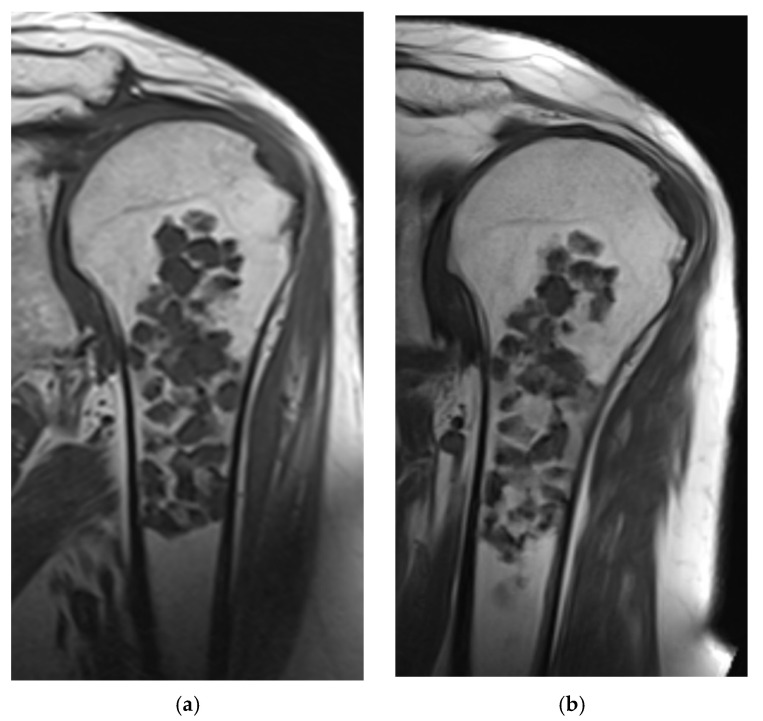
(**a**,**b**): Coronal Spin Echo T1 weighted images of the same cartilaginous tumor three years apart. Signs of fatty replacement are seen peripheral and central in the tumor.

**Figure 4:**
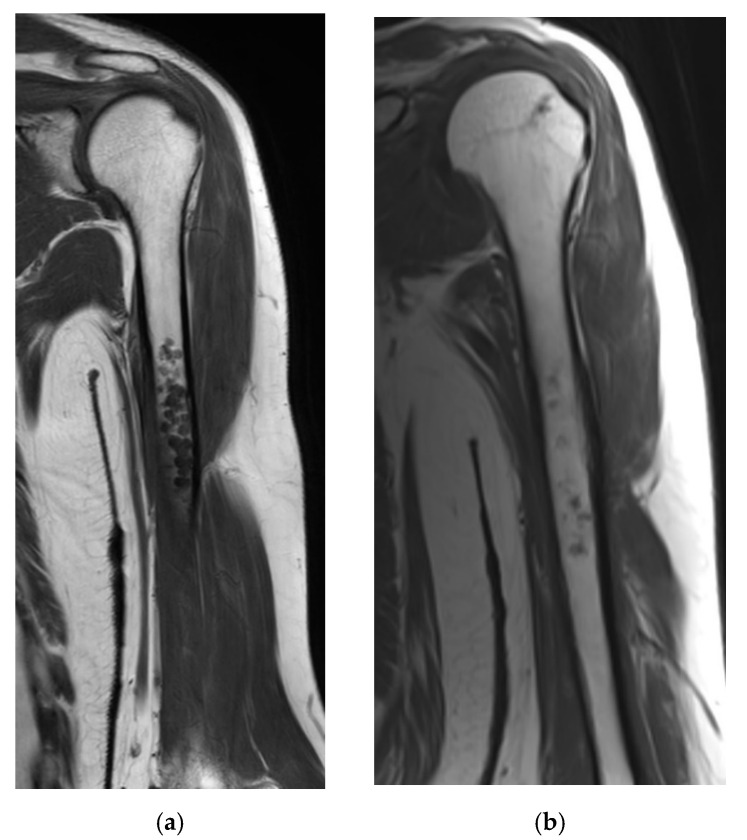
(**a**,**b**): Coronal Spin Echo T1 weighted images of the same cartilaginous tumor four years apart. Almost complete replacement of the cartilaginous tumor by normal bone marrow fat.

Our study provides additional evidence that cartilaginous tumors located in the long bones can be regressive. In the present study, 46 cases (36%) developed signs of regression during follow-up (52 months, range 26–116). In line with Chung et al., we showed a larger incidence of regression than progression during follow-up [12]. Replacement of cartilaginous lobules by normal marrow fat is only mentioned in a few studies, as most studies focus on tumor size, scalloping and development of aggressive features [12,24]. This fatty replacement was seen in 29% of our cases, predominantly in peripheral areas of the tumor (Figure 3 and Figure 4). Sensarma et al. proposed that a process similar to endochondral ossification might explain resorption of cartilage lesions [24]. Entrapped fat was present at diagnosis in majority (87%) of cases that underwent regression and might predict biological behavior. Entrapped fat is known as a reliable MRI characteristic to differentiate benign from malignant tumors [18,19,23,25]. A combination with fatty replacement was seen in 36 of the 90 cases (40%) that either presented with fat entrapment at diagnosis or developed fat entrapment during follow-up. None of the cases in this study showed a decrease of fat entrapment during follow-up. In those six cases of the progression group in which entrapped fat was seen at diagnosis, only minimal growth was reported (median 3.0 mm). Since two cases also showed tumor growth followed by signs of regression, including fat entrapment, we hypothesize that cases with entrapped fat in the progression group might develop signs of regression later on.

All cases that were scored as progressive (*n* = 17; 13%) showed tumor growth, but only in eight cases (6%) tumor growth over 5 mm was seen. The clinical relevance of including cases in the progression group with tumor growth ≤5 mm measured over our midterm follow-up period could be questioned. Although five cases were operated due to tumor growth, surgery could have been redundant to achieve oncological safety, because none showed histopathological signs of HGCS. Besides, it is important to note that tumor progression in this study was seen in relatively small tumors (size at diagnosis ranged from 12 to 64 mm). Cartilage tumors in the long bones smaller than 5 cm are often considered to behave benign and are; therefore, discharged from follow-up in some institutions [26,27]. Based on our findings of tumor growth mainly occurring in relatively small tumors, we would recommend active surveillance irrespective of tumor size. Although patients in the progression group were significantly younger (Table 1), we felt we could not conclude that progression is only seen at young age due to the wide range (20–56 years). Our finding implies that older patients (e.g., >65 years old) might not need frequent follow-up. Future studies are needed to investigate if active surveillance should be tailored to age difference. Previous studies have suggested scalloping to be a distinctive feature to differentiate between enchondroma and ACT [18,28]. The results from this study have shown that presence of scalloping does not predict natural course as scalloping was reported in all three groups and there was no significant difference between these groups (Table 1). The clinical relevance of measuring scalloping could be questioned as there is also only fair observer agreement [3,29].

Active surveillance for ACTs prevents unnecessary surgery and should be the treatment of choice for asymptomatic cartilaginous tumors in the long bones. Due to sparse knowledge on the biological behavior of these tumors, guidelines for active surveillance are lacking and follow-up strategies vary amongst institutions. The Birmingham study group developed a pragmatic imaging tool for triaging cases for referral [11]. We agree that the current rise in incidence of these tumors warrants a pragmatic approach, as a large prospective long term follow-up study would take too long to accomplish. Since we concluded that these tumors show both progression and regression, we propose tumor follow-up schemes should be tailored accordingly.

For active surveillance, we propose an MRI follow-up six months after diagnosis. If HGCS were mistakenly treated with active surveillance, radiologic change of HGCS would be expected within six months. In the study of Davies et al., only two out of 97 patients, with two or more MRI examinations, were diagnosed with HGCS showing malignant characteristics after five and seven months on MRI follow-up [13]. Furthermore, a six-month follow-up would support patients’ wellbeing, since anxiety may arise after the initial diagnosis, which is relieved after a follow-up MRI that shows a stable or regressed tumor.

The next steps in active surveillance are dependent on changes in tumor characteristics at the first follow-up MRI, six months after diagnosis. When the first follow-up shows no changes, a biennial MRI is recommended. This follow-up period might even be extended when the tumor remains stable after longer follow-up. When tumors show regression in asymptomatic patients, we would advise a follow-up MRI at three years after first signs of regression. If the three-year MRI shows no change or continued regression, the patient can be discharged with instructions to contact the hospital in case of new or increased pain. Due to the still limited follow-up of this study, we currently discourage discharge of the patient when first signs of regression are seen. When tumors show progression during follow-up without development of malignant characteristics (e.g., cortical destruction, soft tissue extension), next follow-up MRI is recommended within one year. When tumor growth is accompanied with persistent pain related to the tumor, intralesional surgery is advised.

Although active surveillance seems effective for ACTs in the long bones, we should be cautious for active surveillance to become a cycle of never ending follow-up and prevent overutilization of costly advanced imaging. Therefore, long-term follow-up studies are necessary and predictive nomograms should be developed. Furthermore, future studies should not only focus on aggressive characteristics (e.g., tumor growth, scalloping) but also on characteristics that might predict regression. Based on this study, fat entrapment at diagnosis seems a promising MRI characteristic to predict tumor regression.

This study is limited by its retrospective design, because the indication for surgery changed during the study period. Whereas cases with minimal tumor growth were operated at the start of the study, we currently refrain from surgery. Furthermore, the exact number of enchondroma and ACTs in this active surveillance study is not known as there was no clinical benefit for majority of the patients to perform biopsy or intralesional surgery.

## 5. Conclusions

In conclusion, the natural course of cartilaginous tumors can either show progression, stability or regression. This study shows that active surveillance for enchondroma and ACTs in the long bones is safe, as none of the included tumors developed aggressive characteristics. Follow-up schemes should be tailored according to biological behavior to prevent overutilization of costly advanced imaging.

## Figures and Tables

**Table 1:** Patient demographics.

	No Change (*n* = 65, 51%)	Regression (*n* = 46, 36%)	Progression (*n* = 17, 13%)	*p*-Value
Mean age (range, years)	55 (21–73)	52 (23–76)	39 (20–56)	0.000 ^b, c^
Male: Female	25:40	16:30	9:8	0.419
Median follow-up (months)	46 (25–102)	49 (26–116)	47 (30–138)	0.149
Tumor location (*n*, %)				0.078
Proximal humerus	23 (35.4)	15 (32.6)	3 (17.6)	
Humerus diaphysis	0	4 (8.7)	0	
Proximal femur	12 (18.5)	4 (8.7)	1 (5.9)	
Femur diaphysis	5 (7.7)	7 (15.2)	2 (11.8)	
Distal femur	19 (29.2)	13 (28.3)	7 (41.2)	
Proximal tibia	2 (3.1)	0	2 (11.8)	
Tibia diaphysis	1 (1.5)	1 (2.2)	0	
Distal tibia	0	0	1 (5.9)	
Proximal fibula	2 (3.1)	2 (4.3)	1 (5.9)	
Distal fibula	1 (1.5)	0	0	
Baseline MRI characteristics				
Median size (mm)	38 (13–158)	54 (14–170)	40 (12–64)	0.000 ^a, c^
Excentric location of the tumor	33 (51)	19 (41.3)	6 (35.3)	0.413
Scalloping	34 (52.3)	20 (43.5)	7 (41.2)	0.557
Fat entrapment	40 (61.5)	40 (87.0)	6 (35.3)	0.000 ^a, c^
Septa nodular enhancement	7 (10.8)	4 (8.7)	4 (23.5)	0.107
Calcifications	57 (87.7)	43 (93.5)	13 (76.5)	0.172

Values are displayed as mean/median (range) or *n* (%). ^a^ = significant difference between NC and R, ^b^ = significant difference between NC and P, ^c^ = significant difference between R and P.

**Table 2:** MRI characteristics.

MRI Characteristics	Baseline MRI			Last MRI			
	Present *n* (%)	Absent *n* (%)	N.A.	Increased *n* (%)	Decreased *n* (%)	Unchanged *n* (%)	N.A.
Scalloping	61 (48)	67 (52)	-	0	5 (4)	123 (96)	-
Peritumoral edema	1 (1)	127 (99)	-	0	1 (1)	127 (99)	-
Fat entrapment	86 (67)	42 (33)	-	39 (30)	0	89 (70)	-
Calcifications	113 (88)	15 (12)	-	11 (8)	1 (1)	116 (91)	-
Ring and arc enhancement	116 (91)	0	12	0	20 (16)	94 (73)	14
				**Present**	**Absent**		
Fatty replacement	-	-	-	37 (29)	91 (71)		

N.A.: Not assessable; no contrast enhanced MRI available.

**Table 3:** Operated cases.

Referral	Age at Diagnosis	Location	Initial Size (mm)	Total Growth (mm)	Reason for Surgery	Time in Months from Diagnosis to Surgery	Pathologic Diagnosis
Incidental	49	Tibia (proximal)	30	5	Growth	40	Sample error
Incidental *	47	Femur (distal)	50	11	Growth	43	ACT
Incidental	36	Fibula (proximal)	12	4	Growth	49	ACT
Incidental	22	Femur (distal)	34	23 ^×^	Growth	55	ACT
Incidental *	38	Tibia (distal)	32	7	Growth	53	ACT
Incidental *	64	Femur (distal)	32	-	Prosthesis	57	Enchondroma

* Cases also included in Deckers et al., 2016. ^×^ Growth in combination with loss of ring and arc enhancement on MRI.

## Data Availability

The data presented in this study are available on request from the corresponding author.
